# Kidney Transplant in a 26-Year-Old Nigerian Patient with Sickle Cell Nephropathy

**DOI:** 10.1155/2012/406406

**Published:** 2012-12-03

**Authors:** U. H. Okafor, C. Wachukwu, P. Emem-Chioma, F. S. Wokoma

**Affiliations:** ^1^Renal Unit, Department of Medicine, Enugu State University Teaching Hospital, Enugu, Nigeria; ^2^Renal Unit, Department of Medicine, University of Port Harcourt Teaching Hospital, Port Harcourt, Nigeria

## Abstract

Sickle cell nephropathy (SCN) is a common complication of sickle cell disease (SCD). It has variable presentation, ranging from hyposthenuria to end-stage renal disease (ESRD). Management of ESRD in SCD patients is froth with multiple challenges which has potential to impact negatively the outcome of the patient. Kidney transplant is the preferred renal replacement therapy in these patients. The objective of this case study is to report kidney transplant in a Nigerian young man with sickle cell nephropathy and to highlight the outcome and the challenges to kidney transplant in this patient. The index case is a 26-years-old sickle cell disease patient with ESRD complicated with cardiovascular, pulmonary, immunological, and infective challenges. These conditions were controlled, and the patient had a successful live-related kidney transplant. Kidney transplant is a viable option for sickle cell disease patients with ESRD.

## 1. Introduction

Sickle cell disease (SCD) is a haematological disorder associated with multisystemic complications and manifestations [1]. Sickle cell nephropathy (SCN) leads to both renal functional disturbances and anatomical alterations. The prevalence of renal disease (SCN) in SCA patients is about 25% [2]. The clinical spectrum of SCN include proteinuria, haematuria, nephrotic syndrome, acute renal failure, and chronic progressive renal failure leading to end-stage renal disease (ESRD).

Mechanisms of SCN include fortuitous occurrence, iron overload and its subsequent deposition in the kidney parenchyma, immune complex formations, and focal segmental glomerulosclerosis (FSGS) associated with glomerular hyperfiltration and intrinsic glomerular capillary injury [3, 4].

Treatment of sickle cell nephropathy is fraught with many challenges as a result of antecedent complications prevalent in sickle cell disease patients. The outcome of treatment is usually variable. Renal replacement therapy is inevitable in SCN patients with uraemia, circulatory overload including pulmonary oedema and ESRD. 

Kidney transplant is usually renal replacement therapy of choice for eligible patients with ESRD barring other challenges and limitations. SCD and prevailing complications worsen these challenges, further limiting the prevalence of kidney transplant in these patients. However, in Nigeria and most developing countries kidney transplant is not readily available as a result of some factors which include poverty, ignorance, lack of transplant centres, and lack of donors [5]. There are only 4 kidney transplant centres in Nigeria performing less than 20 kidney transplants per annum. To date there is no documented study or report of kidney transplant in SCD patient in Nigeria. 

## 2. Objective 

The aim of this paper is to report and highlight the outcome of kidney transplant in a 26-year-old Nigerian patient with sickle cell Nephropathy. 

## 3. Case Report

Mr DJ is a 26-year-old student, a male, who was diagnosed with SCA in childhood. He was adherent to his routine medications and regular to follow up. He presented with 2 weeks history of oliguria and passage of frothy urine. This was preceded by swelling of the leg and face a week prior to presentation. This was associated with anorexia, weakness of the body, early satiety, abdominal fullness, and nausea. 

There was no fever, vomiting, jaundice, or change in bowel habit. He had cough productive of frothy sputum but no haemoptysis or chest pain; however, he had dyspnoea, orthopnoea, and paroxysmal nocturnal dyspnoea.

He has had few bone pain crises, and these were usually treated with rehydration, opiate analgesic, antimalarial, and/or antibiotics. He was transfused on 2 occasions as a result of the crises and twice during the course of this illness. He is not a known hypertensive or diabetic and no family history of same or kidney disease. There no positive history of use of herbs, mercury, alcohol, or tobacco. He is the only sickler in a family of 3 siblings; both parents are alive and well. 

He was conscious but in respiratory distress. He had anasarca, pallor, and asterixis but no cyanosis. The pulse was 108 beats/minute, full volume, and regular, the blood pressure was 160/90 mmHg, the praecordium was hyperactive, and apex beat was displaced and heaving. The heart sound heard were S1, S2, and S3 gallop with pansystolic murmur maximal at the apex. 

He had bilateral basal fine crepitations, distended abdomen with firm smooth hepatomegaly and ascites. He was conscious and had no asterixis. 

He was admitted as a case of sickle cell cardiomyopathy in congestive cardiac failure, with sickle cell nephropathy as a differential diagnosis. 

 Investigations results revealed that urinalysis showed proteinuria 3+. PCV was 14%, blood film hypochromia, polychromasia, anisocytosis, and fragmented cells. The total and differential white blood cell count was normal, and ESR was 94 mm/hr. The serum sodium was 132 mmol/L, potassium was 4.1 mmol/L, bicarbonate was 13 mmol/L, creatinine was 645 *μ*mol/L, urea was 35 mmol/L, calcium was 2.3 mmol/L, total protein was 59 mg/dL, albumin was 28 mg/dL, total cholesterol was 5.2 mmol/L, LDL was 3.2 mmol/L, and HDL was 1.1 mmol/L.

The abdominal ultrasound showed bilateral shrunken kidneys, hepatomegaly, and ascites. Other viscera were normal. 

Chest X-ray showed marked cardiomegaly, pulmonary oedema as abnormality.

Echocardiography revealed marked 4-chamber dilatation and biventricular failure.

The diagnosis was then changed to sickle cell nephropathy with congestive cardiac failure. He was comanaged with the cardiologist.

He was advised on low salt diet, care on protein intake, and was commenced on tabs Ramipril, hydrochlorothiazide, frusemide, digoxin, erythropoietin, folic acid, proguanil, vasoprine, and erythropoietin. He was also on twice weekly haemodialysis. He was transfused with 2 units of packed red blood cell.

The haemodialysis including ultrafiltration and medication was regular; however, the breathlessness and ascites persisted. Weekly therapeutic abdominal paracentesis for symptomatic relief was then instituted while awaiting result of other investigations.

Results of further investigations revealed sterile exudative ascitic fluid. Chest CT scan revealed enlarged heart, mild pericardial effusion, and prominent main pulmonary artery. Abdominal CT scan revealed bilateral small kidneys, gross ascites, hepatomegaly, cholelithiasis, dilatation of the portal vein, and prominent collaterals seen at the porta hepatis. BACTEC blood culture was positive for tuberculosis. Other investigations were consistent with previous results as documented above.

The managing team then was expanded and included nephrologists, infectious disease physician, cardiologist, gastroenterologist, and haematologist.

The frequency of haemodialysis was increased to daily, antituberculous therapy was commenced, and tabs Sildenafil were added to the medications above. He improved remarkably within 2 weeks of these regimens. 

The patient improved progressively, and 4 weeks later he had a successful and live-related kidney transplant. There was no operative or immediate postoperative complication. He is 1-year posttransplant, there was complication, and he has remained stable clinically, biochemically, and haematologically. His present blood pressure was 120/70 mmHg, PCV was 25%, sodium was 137 mmol/L, potassium was 4.1 mmol/L, Serum creatinine was 96 umol/L, serum urea was 5.3 mmol/L, and tacrolimus level was 4.8 ng/L.

## 4. Discussion

Sickle cell disease (SCD) patients with end-stage renal disease (ESRD) can be treated with all forms of renal replacement therapy: hemodialysis, peritoneal dialysis, or kidney transplant. Our patient was treated initially with haemodialysis and later had kidney transplant. Earlier reports suggested poor allograft survival and other disease-specific problems in SCD patients who had kidney transplant [6]. However, more studies had reported graft and patient survival rates comparable to those of other nondiabetic patients [7–9]. 

There was a trend toward improved survival in those SCD patients who received transplants compared to those on chronic dialysis. One-year patient and graft survival were 87 and 67%, respectively [9]. In an update registry in 1987, data recollected from 45 renal transplants performed in 40 recipients revealed a one-year patient survival rate of 88%. The graft survival rate at one year was 82% in living donor transplant recipients and 62% in cadaveric transplant recipients [7].

Ojo and colleagues [8] reported that the short-term survival of renal allograft in recipients with end-stage sickle cell nephropathy (SCN) was similar to that achieved in patients with other causes of end-stage renal disease, but the long-term outcome was comparatively diminished. 

Our patient had a remarkable improvement following the transplant. There was no operative, postoperative patient, or graft-related complication. He is a year posttransplant, and there had not been any notable complication. The graft is structurally and functionally normal. The patient is clinically stable, and the blood pressure and haematological parameters were normal. There was no sickle crisis since the kidney transplant. And the patient had recommenced his education. 

Despite encouraging results on survival advantage of renal transplantation over maintenance haemodialysis, renal transplantation in sickle cell disease is used less frequently, possibly reflecting the limited experience with renal transplantation and/or the various challenges associated with kidney transplant in this patient population. 

Although many SCD patients as documented above including our patient have done well after renal transplantation, several unique challenges and complications have been described [7]. The primary abnormality of SCD patient is the presence of sickle cell in their peripheral blood. This causes impairment in the perfusion of the tissues and had led to injury and occasionally failure of various organs. This predisposes the patient to various organ dysfunctions that can delay or preclude kidney transplant or encourage complication following kidney transplant in this patient. 

Patients with sickle cell nephropathy are at risk of developing various haematologic, cardiovascular, pulmonary, and immunologic complications. These influence and compromise the choice, fitness, and outcome of kidney transplant in these patients. The impact of these conditions could manifest during pretransplant work-up, surgery, or posttransplant management. Various authors [10, 11] have reported that the risk of morbidity and mortality in these patients is greater than in the general population because of anemia, the propensity for red blood cells to sickle and obstruct the microvasculature, the presence of chronic organ damage in some patients, the risks of hypoxia, and the effects of asplenia. Sickle cell disease patients are subject to other medical problems, including impaired physical development, hepatic, gall bladder, gum disease, and multiple organ failure. These conditions can pose challenges in preoperative, operative, and postoperative management of kidney transplant in SCD patients.

Our patient presented with various challenging features which included pulmonary oedema, pulmonary hypertension, cardiomyopathy, pericardiac effusion, cardiac failure, portal hypertension, marked ascities, abdominal tuberculosis, autosplenectomy, and multiple blood transfusion. These challenges are worrisome; it ranges from anaesthetic/surgical risk, difficulty in controlling pre- and posttransplant infection, marked immunosuppression and alloimmunization, and its impact in obtaining compatible donor to poor wound healing. 

Various suggestions for risk reduction have been made, including correction of anemia by simple or exchange blood transfusion, attention to hydration and oxygenation, postoperative respiratory care, and selection of less aggressive or extensive surgical procedures. The protocol specified a minimum of 8 hours of preoperative hydration, with intraoperative monitoring of temperature, blood pressure, electrocardiographic features, and oxygenation. Postoperative care included the administration of oxygen, intravenous hydration, monitoring with pulse oximetry, and respiratory therapy [11]. The patients need to be nursed in the intensive care unit with strict monitoring and prompt intervention by well-trained and dedicated team.

Bone marrow transplantation has recently emerged as a novel treatment for SCD. Anecdotal reports suggest a beneficial effect of bone marrow transplantation in improving target organ damage, including chronic lung, bone, and central nervous system disease. Whether bone marrow transplantation in the early stage of the disease can reverse or halt the progression of established sickle cell nephropathy is unknown and awaits clinical studies. Further more experience in stem cell and kidney transplant is lacking; however, it may have potential in improving the long-term outcome of kidney transplant in sickle cell patients [12].

As noted above, the patient reported improved significantly on management of the prevailing challenging clinical condition he presented with and had uneventful kidney transplant. This could be attributed to adequate interventions taken in this patient including the multidisciplinary approach to combat the challenges with daily haemodialysis, therapy for infection, reduction of pulmonary and portal hypertension, induction immunosuppression, and extended nursing in intensive care unit. 

Patients with sickle cell disease requiring kidney transplant are also prone to challenges experienced by their normal counterpart. This varies from universal challenges like scarcity of donor to challenges peculiar to resource poor nations like poverty, ignorance, lack of facilities, and personnel [5].

In conclusion kidney transplant in patients with sickle cell nephropathy is plagued with multiplicity of challenges; however, outcome is encouraging and comparable to that of patients with normal haemoglobin AA. There is need for multidisciplinary and subspecialty coordination in management of these patients. Thus sickle cell disease or trait should not be regarded as a contraindication to transplantation, at least from the patient and allograft survival standpoint.
